# Genetic variants in *CYP4F2* were significantly correlated with susceptibility to ischemic stroke

**DOI:** 10.1186/s12881-019-0888-6

**Published:** 2019-09-11

**Authors:** Yuan Wu, Junjie Zhao, Yonglin Zhao, Tingqin Huang, Xudong Ma, Honggang Pang, Ming Zhang

**Affiliations:** 1 Department of Critical Care Medicine, the Second Affiliated Hospital of Xi’an Jiaotong University, Xi’an, 710004 Shaanxi China; 2 Department of Neurosurgery, the First Affiliated Hospital of Xi’an Jiaotong University, Xi’an, 710061 Shaanxi China; 3 Department of Oncology Hospital, the Second Affiliated Hospital of Xi’an Jiaotong University, Xi’an, 710004 Shaanxi China; 4 Department of Neurosurgery, the Second Affiliated Hospital of Xi’an Jiaotong University, Xi’an, 710004 Shaanxi China; 5 Department of Surrounding Vascular, the First Affiliated Hospital of Xi’an Jiaotong University, Xi’an, 710061 Shaanxi China

**Keywords:** Ischemic stroke (IS), Cytochrome P450 family 4 subfamily F member 2 (*CYP4F2*), Case-control study, Single nucleotide polymorphisms (SNPs)

## Abstract

**Background:**

Ischemic stroke (IS) is a serious cardiovascular disease and is associated with several single nucleotide polymorphisms (SNPs). However, the role of Cytochrome P450 family 4 subfamily F member 2 (*CYP4F2*) gene in IS remains unknown. Our study aimed to explore whether *CYP4F2* polymorphisms influenced IS risk in the Han Chinese population.

**Methods:**

We selected 477 patients and 495 controls to do a case-control study, and five SNPs in *CYP4F2* gene were successfully genotyped. And we evaluated the associations using the Chi-squared test, independent sample t test, and genetic models analyses. Logistic regression analysis was used to calculate odds ratios (ORs) and 95% confidence intervals (CIs).

**Results:**

In this study, rs12459936 and rs3093144 were associated with IS risk in the overall. After stratified analysis by age (> 61 years), rs3093193 and rs3093144 were related to an increased risk of IS, whereas rs12459936 was related to a decreased risk of IS. In addition, we found that three SNPs (rs3093193, rs3093144 and rs12459936) were associated with the susceptibility to IS in males. We also found five SNPs in the *CYP4F2* gene had strong linkage.

**Conclusions:**

Three SNPs (rs3093193, rs3093144 and rs12459936) in the *CYP4F2* were associated with IS risk in a Chinese Han population. And, *CYP4F2* gene may be involved in the development of IS.

**Supplementary information:**

**Supplementary information** accompanies this paper at (10.1186/s12881-019-0888-6).

## Background

Stroke is the leading neurological cause of death worldwide [1]. It is also called brain attack, typically characterized by neurological dysfunction due to acute focal injury of the central nervous system [2]. At a given age, the incidence and mortality rates of stroke are higher in men, however, women are more likely to have stroke because of longer life, and stroke events increase rapidly with aging [3]. Moreover, stroke is already becoming a global issue and it is threatening the health of human beings. Additionally, epidemiologic studies reported that the incidence of IS in China is significantly higher than that in the developed countries [4].

Hence, it is urgent to clarify the etiology of stroke. The majority of stroke are ischemic (approximately 80%), whereas 20% is due to primary hemorrhage [5]. Several studies demonstrated that some risk factors are involved in the development of ischemic stroke (IS), such as a family history of cardiovascular disease, older age, sex [6, 7], hypertension [8], diabetes [9]. However, a large proportion of IS remains unclear. Recently, the role of genetic factors in development of IS was determined in large-scale, collaborative, genome-wide association studies (GWAS) [10]. Some susceptibility genes of IS have been found, including *PITX2* [11], *ABO* [12], and *HABP2* [13]. It demonstrated that genetic factors may regulate the pathophysiological process of IS.

Cytochrome P450 (CYP450) plays a vital role in the metabolism of exogenous and endogenous compounds and is widely distributed in human [14]. Cytochrome P450 family 4 subfamily F member 2 (*CYP4F2*) as a member of CYP450 superfamily, is located on human chromosome 19p13.11 [15]. Previous studies have demonstrated *CYP4F2* gene polymorphisms are associated with the risk of IS. For example, Nakamura et al. [16] conducted a case-control study based on the Japanese population to identify whether single nucleotide polymorphisms (SNPs) in *CYP4F2* gene associated with the susceptibility to IS, they found ‘G’ allele of rs2108622 was correlated with increased risk of IS in male patients. A study in Sweden [17] reported the V433 M mutation in the *CYP4F2* was correlated with cerebral infarction in male patients. It provided strong evidence on the association of *CYP4F2* gene and IS. In the present study, we selected five candidate SNPs (rs3093203, rs3093193, rs12459936, rs3093144, and rs3093110) in *CYP4F2* and performed a case-control study in Han Chinese population.

## Methods

### Subjects

The 477 cases were recruited from the First and Second Affiliated Hospital of Xi’an Jiaotong University between January 2016 and October 2018. In our study, all cases were consistent with the World Health Organization’s diagnostic criteria for IS. IS was confirmed by at least two independent neurologists using computed tomography (CT) scans and/or magnetic resonance imaging (MRI). IS patients with history of transient ischemic attack, coronary artery disease, autoimmune disease, systemic inflammatory disease, malignant tumor and other chronic diseases were excluded from the study. Healthy individuals (495 controls) were identified through the annual health assessment and were randomly recruited from the same hospital physical examination center between April 2016 and October 2018. All members were Han Chinese population living in the Shaanxi Province of China and were unrelated in at least three generations.

### SNP selection and genotyping

In total, we successfully select five variants (rs3093203, rs3093193, rs12459936, rs3093144, and rs3093110) in the global population of the 1000 genome (http://www.internationalgenome.org/) with minor allele frequencies (MAFs) > 5%. Blood samples were collected in the test tubes containing ethylenediamine tetraacetic acid (EDTA) for anticoagulation and stored at − 80 °C after centrifugation at 2000 rpm for 10 min. Genomic DNA was extracted from peripheral blood samples of the 477 cases and the 495 controls using genomic DNA purification kit (GoldMag, Xi’an, China). And we used NanoDrop 2000 (Thermo Scientific, Waltham, MA) to measure the DNA concentration and purity. Agena MassARRAY Assay Design 3.0 Software [18] was used to design the primers for amplification and extension reactions. The information of primers was shown in Table 1. We used Agena MassARRAY RS1000 to perform the SNP genotyping according to the manufacturer’s instruction and used Agena Typer 4.0 software to manage and analyze the data [18, 19].

**Table 1:** Primers used for this study

SNP_ID	1st-PCR primer	2nd-PCR primer	UEP SEQ
rs3093203	ACGTTGGATGAAAGCCACCAATCCGCTATG	ACGTTGGATGGGTCACATAGTGTACTGTCC	ACATAGTGTACTGTCCTTTTATA
rs3093193	ACGTTGGATGGTGATGAGACTAGTGATCCC	ACGTTGGATGGCCACATACACATTGATGGG	GTTTAGATAAACAGCCACA
rs12459936	ACGTTGGATGGGTAACCATCATTCTGCTTC	ACGTTGGATGAGAGGTCGCAGTAAGCTGAG	CAGCCTGGGTGACAGAG
rs3093144	ACGTTGGATGGGGAAGAATTGTGGCAAAGG	ACGTTGGATGAGGAGTCTCTCGTCCTTCTG	AGTTAAAAAAAAAATCCTAGATACTT
rs3093110	ACGTTGGATGGTCTCATTGATAAGAGGGAG	ACGTTGGATGTCCTGTTATGAGGGTACAGC	CCGTCTCCCACTTCCAC

*SNP* single nucleotide polymorphism, *UEP SEQ* Unextended mini-sequencing primer

### Statistical analysis

Excel (Microsoft Corp., Redmond, WA, United States) and SPSS statistical software (version 17.0, SPSS, Chicago, IL) were used for statistical analyses. All statistical tests were two-sided, and *p* < 0.05 was considered to be statistical significance. SNP genotype frequencies among cases and controls were calculated by Chi- squared test, and the Hardy–Weinberg equilibrium (HWE) was used to compare the actual and expected frequencies of the genotypes in the controls. In order to explore the effects of the *CYP4F2* variants on the risk of IS based on the various genetic models (co- dominant, dominant, recessive, and log- additive), logistic regression analysis was used to calculate the odds ratios (ORs) and 95% CIs on PLINK software (version 1.07) [20]. Finally, the Haploview software package (version 4.2) and SNPStats (https://www.snpstats.net/start.htm?q=snpstats/start.htm) were used to estimate pairwise linkage disequilibrium (LD), haplotype construction, and genetic association at polymorphism loci [21, 22]. HaploReg v4.1 (https://pubs.broadinstitute.org/mammals/haploreg/haploreg.php) was used to predict the potential functions of variants in *CYP4F2* gene.

## Results

### Study participants

The basic characteristics of all study participants were shown in Table 2. Four hundred seventy-seven cases (316 males and 161 females) and 495 healthy controls (326 males and 169 females) were recruited in this case-control study. The mean ages of the patients and the controls were 60.05 ± 6.56 and 64.13 ± 10.82 years old, respectively. There was no statistically significant difference (*p* = 0.898) on the gender distribution, while the age distribution was statistically significant difference (*p* = 0.000). As expected, the prevalence of the most common risk factor for IS were significantly different between cases and controls, such as HDL cholesterol and LDL cholesterol (*p* < 0.001).

**Table 2:** Basic characteristics of controls and cases

Characteristics	Cases N (%)	Controls N (%)	*p*-value
Number	477	495	
Age,year (mean ± SD)	60.05 ± 6.56	64.13 ± 10.82	0.000^a^
> 61	274 (57.4%)	212 (42.8%)	
≤61	203 (42.6%)	283 (57.2%)	
Gender, no, %			0.898^b^
Male	316 (66.2%)	326 (65.9%)	
Female	161 (33.8%)	169 (34.1%)	
Triglycerides (mmol/L)	1.78 ± 1.39	1.59 ± 1.06	0.051^a^
Cholesterol (mmol/L)	4.39 ± 1.20	3.93 ± 0.88	0.331 ^a^
HDL-C (mmol/L)	0.92 ± 0.47	1.09 ± 0.26	**< 0.001** ^a*^
LDL-C (mmol/L)	2.11 ± 1.76	1.81 ± 0.81	**< 0.001** ^a*^

^a^*p* values were calculated from Student t tests

^b^*p* values were calculated by two-sided Chi-square tests

*Bold values indicate statistical significance (*p* < 0.05)

### The association between CYP4F2 gene and the risk of IS

Table 3 summarized the basic information of the SNPs including gene, SNP ID, chromosomal position, minor allele frequency (MAF) of cases and controls and HWE test results. Five SNPs in the *CYP4F2* gene were successfully genotyped for further analysis. The genotype distribution of all SNPs conformed to HWE in control subjects (*p* > 0.05). The differences in allele frequency distributions between patients and controls were identified by two-sided Pearson chi-squared tests. The rs12459936 was associated with decreased risk of IS (OR = 0.82, 95% CI = 0.68–0.98, *p =* 0.028). And the rs3093144 was associated with 1.27-fold (95% CI = 1.01–1.60, *p* = 0.043) increase in the risk of IS. Further, multiple inheritance models were applied for analyzing the association between *CYP4F2* SNPs and IS risk by unconditional logistic regression analysis adjusted for age and gender (Table 4).

**Table 3:** Basic information of candidate SNPs in *CYP4F2* and associations with stroke

SNP-ID	Chr	Gene	Position	Alleles	MAF		*p*^a^-HWE	OR (95% CI)	*p*^b^-value
				(minor/major)	cases	controls			
rs3093203	19	*CYP4F2*	15,878,374	A/G	0.235	0.227	0.608	1.04 (0.85–1.29)	0.691
rs3093193	19	*CYP4F2*	15,881,104	G/C	0.324	0.287	0.584	1.19 (0.98–1.44)	0.081
rs12459936	19	*CYP4F2*	15,882,231	T/C	0.412	0.462	0.470	0.82 (0.68–0.98)	0.028*
rs3093144	19	*CYP4F2*	15,891,487	T/C	0.199	0.164	0.621	1.27 (1.01–1.60)	0.043*
rs3093110	19	*CYP4F2*	15,896,974	G/A	0.127	0.126	0.839	1.01 (0.77–1.32)	0.930

*SNP* single nucleotide polymorphism, MAF: minor allele frequency, *HWE* Hardy-Weinberg equilibrium, *OR* odds ratio, *95% CI*: 95%confidence interval

*p*^a^-value were calculated from Fisher’s exact test; ^b^ *p* values were calculated from two-sided χ^2^ test

*Bold values indicate statistical significance (*p* < 0.05)

**Table 4:** Association of SNPs with risk of stroke based on logistic tests adjusted by gender and age

SNP-ID	Model	Genotype	No. (frequency)		Adjusted^a^	
			Case	Control	OR (95% CI)	*p*^b^-value
rs3093203	codominant	G/G	275 (57.7%)	292 (59.2%)	1.00	–
		A/G	180 (37.7%)	178 (36.1%)	1.07 (0.82–1.41)	0.614
		A/A	22 (4.6%)	23 (4.7%)	0.90 (0.48–1.69)	0.737
	dominant	G/G	275 (57.7%)	292 (59.2%)	1.00	–
		A/G-A/A	202 (42.3%)	201 (40.8%)	1.02 (0.82–1.27)	0.868
	recessive	G/G-A/G	455 (95.4%)	470 (95.3%)	1.00	–
		A/A	22 (4.6%)	23 (4.7%)	1.05 (0.81–1.37)	0.706
	log-additive	–	–	–	0.87 (0.47–1.63)	0.670
rs3093193	codominant	C/C	216 (45.3%)	248 (50.2%)	1.00	–
		G/C	213 (44.7%)	208 (42.1%)	1.14 (0.87–1.50)	0.342
		G/G	48 (10.01)	38 (7.7%)	1.39 (0.86–2.23)	0.178
	dominant	C/C	216 (45.3%)	248 (50.2%)	1.00	–
		G/C-G/G	261 (54.7%)	246 (49.8%)	1.16 (0.95–1.42)	0.143
	recessive	C/C-G/C	429 (89.9%)	456 (92.3%)	1.00	–
		G/G	48 (10.1%)	38 (7.7%)	1.18 (0.91–1.53)	0.214
	log-additive	–	–	–	1.30 (0.82–2.05)	0.259
rs12459936	codominant	C/C	158 (33.5%)	139 (28.1%)	1.00	–
		T/C	238 (50.5%)	254 (51.4%)	0.89 (0.66–1.19)	0.422
		T/T	75 (16.0%)	101 (20.5%)	0.71 (0.48–1.05)	0.082
	dominant	C/C	158 (33.5%)	139 (28.1%)	1.00	–
		T/C-T/T	313 (66.5%)	355 (61.9%)	0.85 (0.70–1.03)	0.089
	recessive	C/C-T/C	396 (84.0%)	393 (79.5%)	1.00	–
		T/T	75 (16.0%)	101 (20.5%)	0.84 (0.63–1.11)	0.212
	log-additive	–	–	–	0.77 (0.55–1.08)	0.123
rs3093144	codominant	C/C	304 (64.0%)	344 (69.5%)	1.00	–
		T/C	153 (32.2%)	140 (28.3%)	1.18 (0.88–1.56)	0.266
		T/T	18 (3.8%)	11 (2.2%)	1.95 (0.89–4.28)	0.095
	dominant	C/C	304 (64.0%)	344 (69.5%)	1.00	–
		T/C-T/T	171 (36.0%)	151 (30.5%)	1.24 (0.98–1.58)	0.075
	recessive	C/C-T/C	457 (96.2%)	484 (97.8%)	1.00	–
		T/T	18 (3.8%)	11 (2.2%)	1.23 (0.93–1.62)	0.142
	log-additive	–	–	–	1.86 (0.85–4.05)	0.120
rs3093110	codominant	A/A	364 (76.3%)	378 (76.5%)	1.00	–
		G/A	105 (22.0%)	108 (21.9%)	1.01 (0.74–1.37)	0.977
		G/G	8 (1.7%)	8 (1.6%)	1.00 (0.37–2.75)	0.994
	dominant	A/A	304 (64.0%)	344 (69.5%)	1.00	–
		G/A-G/G	171 (36.0%)	151 (30.5%)	1.00 (0.76–1.32)	0.978
	recessive	A/A-G/A	469 (98.3%)	486 (98.4%)	1.00	–
		G/G	8 (1.7%)	8 (1.6%)	1.01 (0.74–1.36)	0.977
	log-additive	–	–	–	1.00 (0.37–2.74)	0.996

Adjusted^a^ for age and sex in a logistic regression model

*p*^b^ values were calculated from wald test

*p* values indicate statistical significance (*p* < 0.05)

### Stratification analysis by age and gender

Next, we conducted stratified analysis by age. The results showed that three SNPs were associated with the risk of IS in patients (age > 61 years old), as displayed in Table 5. Rs3093193 in *CYP4F2* gene increased the susceptibility of IS in allele model (G vs. C, OR = 1.34, 95% CI = 1.02–1.77, *p* = 0.038), dominant model (GC/GG vs. CC, OR = 1.61, 95% CI = 1.07–2.42, *p* = 0.022) and log-additive model (OR = 1.44, 95% CI = 1.05–1.98, *p* = 0.024). Similarly, rs3093144 was observed to be associated with the higher risk of IS in allele model (T vs. C, OR = 1.53, 95% CI = 1.09–2.14, *p* = 0.013), dominant model (TC/TT vs. CC, OR = 1.62, 95% CI = 1.05–2.49, *p* = 0.029) and log-additive model (OR = 1.56, 95% CI = 1.07–2.27, *p* = 0.020). Conversely, rs12459936 was observed to be associated with the decreased risk of IS in allele model (T vs. C, OR = 0.73, 95% CI = 0.56–0.94, *p* = 0.016), co-dominant model (TT vs. CC, OR = 0.48, 95% CI = 0.26–0.88, *p* = 0.018), dominant model (TC/TT vs. CC, OR = 0.63, 95% CI = 0.41–0.94, *p* = 0.036) and log-additive model (OR = 0.69, 95% CI = 0.52–0.93, *p* = 0.015). In addition, these was no other significant association between SNPs and IS risk (age ≤ 61 years).

**Table 5:** Stratification analysis of the association of *CYP4F2* polymorphisms with stroke under genetic models

SNP	Model	Genotype	> 61		≤61		Male		Female	
			OR(95%CI)	*p-*value	OR(95%CI)	*p*-value	OR(95%CI)	*p*-value	OR(95%CI)	*p*-value
rs3093193	allele	C	1.00		1.00		1.00		1.00	
		G	**1.34 (1.02–1.77)**	**0.038** ^*^	1.04 (0.79–1.37)	0.791	**1.35 (1.06–1.71)**	**0.016** ^*^	0.95 (0.69–1.32)	0.757
	co-dominant	CC	1.00	–	1.00	–	1.00	–	1.00	–
		GC	1.56 (1.02–2.39)	0.101	1.09 (0.74–1.61)	0.657	1.10 (0.79–1.54)	0.567	1.21 (0.76–1.92)	0.421
		GG	1.86 (0.89–3.92)	**0.04**0	1.27 (0.64–2.50)	0.494	**2.29 (1.21–4.32)**	**0.011** ^*^	0.64 (0.29–1.41)	0.267
	dominant	C/C	1.00	–	1.00	–	1.00	–	1.00	–
		G/C-G/G	**1.61 (1.07–2.42)**	**0.022** ^*^	1.12 (0.77–1.63)	0.551	1.24 (0.90–1.71)	0.195	1.08 (0.70–1.68)	0.731
	recessive	C/C-G/C	1.00	–	1.00	–	1.00	–	1.00	–
		G/G	1.50 (0.73–3.05)	0.268	1.21 (0.63–2.33)	0.560	**2.19 (1.18–4.05)**	**0.013** ^*^	0.58 (0.27–1.23)	0.157
	log-additive	–	**1.44 (1.05–1.98)**	**0.024** ^*^	1.11 (0.83–1.49)	0.472	**1.31 (1.02–1.69)**	**0.036** ^*^	0.93 (0.67–1.31)	0.693
rs12459936	allele	C			1.00		1.00		1.00	
		T	**0.73 (0.56–0.94)**	**0.016** ^*^	0.96 (0.74–1.23)	0.727	**0.78 (0.63–0.98)**	**0.031** ^*^	0.89 (0.65–1.21)	0.449
	co-dominant	CC	1.00	–	1.00	–	1.00	–	1.00	–
		TC	0.70 (0.44–1.10)	0.117	1.13 (0.73–1.75)	0.582	0.94 (0.64–1.36)	0.729	0.80 (0.49–1.31)	0.383
		TT	**0.48 (0.26–0.88)**	**0.018** ^*^	0.79 (0.45–1.38)	0.406	0.65 (0.40–1.05)	0.079	0.86 (0.45–1.62)	0.637
	dominant	C/C	1.00	–	1.00	–	1.00	–	1.00	–
		T/C-T/T	**0.63 (0.41–0.97)**	**0.036** ^*^	1.03 (0.68–1.56)	0.898	0.85 (0.60–1.22)	0.380	0.82 (0.52–1.30)	0.393
	recessive	C/C-T/C	1.00	–	1.00	–	1.00	–	1.00	–
		T/T	0.59 (0.34–1.03)	0.060	0.73 (0.45–1.17)	0.189	0.68 (0.44–1.03)	0.068	0.97 (0.55–1.72)	0.922
	log-additive	–	**0.69 (0.52–0.93)**	**0.015** ^*^	0.91 (0.69–1.19)	0.493	0.82 (0.65–1.04)	0.103	0.91 (0.66–1.24)	0.529
rs3093144	allele	C	1.00		1.00		1.00		1.00	
		T	**1.53 (1.09–2.14)**	**0.013** ^*^	1.06 (0.76–1.48)	0.741	**1.36 (1.01–1.82)**	**0.040** ^*^	1.13 (0.77–1.66)	0.520
	co-dominant	CC	1.00	–	1.00	–	1.00	–	1.00	–
		TC	1.54 (0.98–2.41)	0.059	1.03 (0.68–1.55)	0.890	1.15 (0.81–1.64)	0.438	1.24 (0.77–2.00)	0.383
		TT	2.57 (0.80–8.25)	0.114	1.75 (0.58–5.31)	0.324	**4.5 (1.22–16.64)**	**0.024** ^*^	0.93 (0.31–2.81)	0.902
	dominant	C/C	1.00	–	1.00	–	1.00	–	1.00	–
		T/C-T/T	**1.62 (1.05–2.49)**	**0.029** ^*^	1.08 (0.73–1.60)	0.569	1.25 (0.89–1.77)	0.198	1.20 (0.76–1.89)	0.445
	recessive	C/C-T/C	1.00	–	1.00	–	1.00	–	1.00	–
		T/T	2.25 (0.70–7.17)	0.171	1.73 (0.58–5.22)	0.328	**4.32 (1.17–15.89)**	**0.028** ^*^	0.87 (0.29–2.60)	0.807
	log-additive	–	**1.56 (1.07–2.27)**	**0.020** ^*^	1.12 (0.79–1.58)	0.522	1.34 (0.98–1.82)	0.067	1.11 (0.76–1.64)	0.579

*p* values were calculated from wald test

*Bold values indicate statistical significance (*p* < 0.05)

Moreover, we conducted stratified analysis by gender and found three loci associated with the risk of IS in male. Two of these SNPs were correlated with the increased risk of IS in allele model (rs3093193, G vs. C, OR = 1.35, 95% CI = 1.06–1.71, *p* = 0.016; rs3093144, T vs. C, OR = 1.36, 95% CI = 1.01–1.82, *p* = 0.040), co-dominant model (rs3093193, GG vs. CC, OR = 2.29, 95% CI = 1.21–4.32, *p* = 0.011; rs3093144, TT vs. CC, OR = 4.5, 95% CI = 1.22–16.64, *p* = 0.024), recessive model (rs3093193, GG vs. CC/GC, OR = 2.19, 95% CI = 1.18–4.05, *p* = 0.013; rs3093144, TT vs. CC/TC, OR = 4.32, 95% CI = 1.17–15.89, *p* = 0.028) and log-additive model (rs3093193, OR = 1.31, 95% CI = 1.02–1.69, *p* = 0.036). Conversely, rs12459936 was associated with decreased risk of IS in allele model (T vs. C, OR = 0.78, 95% CI = 0.63–0.98, *p* = 0.031).

### Associations between haplotype analyses and IS risk

**Fig. 1:**
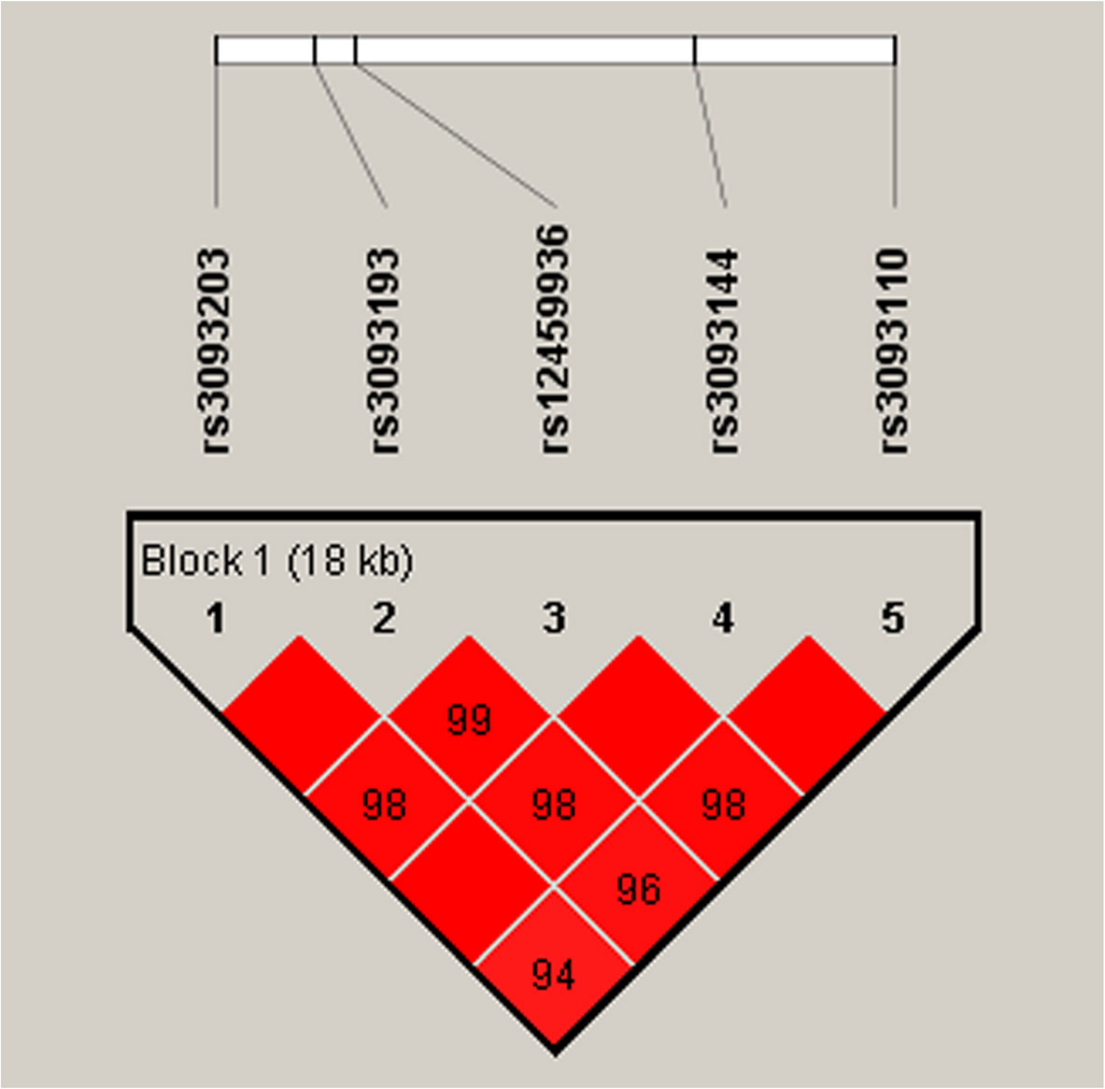
Linkage disequilibrium (LD) analysis of five SNPs in *CYP4F2* Gene

The LD and haplotype analyses of the *CYP4F2* polymorphisms in the cases and controls were further studied. All SNPs in *CYP4F2* gene were found to exist in LD block (Fig. 1). The distributions of different haplotypes in two groups are presented in Table 6. Although the five SNPs in the *CYP4F2* gene had strong linkage, haplotype analysis did not show the significant association (*p* > 0.05). Then, we conducted haplotype analysis of three positive SNPs (rs3093193, rs12459936 and rs3093144) and IS risk (Table 7**)**. The result demonstrated that the haplotype “GCT” was linked to an increased risk of IS (OR = 1.31, 95% CI = 1.01–1.70, *p* = 0.043).

**Table 6:** Haplotype frequencies of *CYP4F2* SNPs and the association with stroke

Block	Haplotype	Frequency		OR (95% CI)	*p-*value
		Case (477)	Control (495)		
Block 1	rs3093203|rs3093193|rs12459936|rs3093144|rs3093110				
	GGCCG	418 (87.7%)	436 (88%)	0.99 (0.75–1.31)	0.944
	GGCTA	94 (19.8%)	80 (16.1%)	1.25 (0.99–1.60)	0.065
	GCTCA	280 (58.8%)	268 (54.1%)	1.17 (0.97–1.41)	0.109
	ACCCA	111 (23.2%)	111 (22.4%)	1.02 (0.82–1.28)	0.837
	GCCCA	465 (97.4%)	483 (97.5%)	1.01 (0.57–1.81)	0.970

OR: odds ratio; 95%CIs: 95% confidence intervals

*p* values were calculated by logistic regression with adjustment for age and gender

*p* values indicate statistical significance (*p* < 0.05)

**Table 7:** Haplotype frequencies of *CYP4F2* positive SNPs and the association with stroke

Block	Haplotype	Frequency		OR (95% CI)	*p-*value
		Case(477)	Control(495)		
Block 1	rs3093193/rs12459936/rs3093144				
	CTC	196 (41.1%)	228 (46%)	1	–
	CCC	124 (26.1%)	125 (25.2%)	1.12 (0.88–1.41)	0.360
	GCT	94 (19.7%)	80 (16.2%)	**1.31 (1.01–1.70)**	**0.043** ^*^
	GCC	(61 (12.7%)	61 (12.3%)	1.11 (0.83–1.49)	0.490

*OR* odds ratio, *95% CIs* 95% confidence intervals

*Bold values indicate statistical significance (*p* < 0.05)

### In silico analysis

In silico analysis, HaploReg v4.1 was used to assess the function of the selected variants in *CYP4F2* gene (Additional file 1: Table S1). The results suggested rs3093203, rs3093193, and rs12459936 were associated with Motifs changed, selected eQTL hits. And rs3093144 was associated with enhancer histone marks, motifs changed, selected eQTL hits. Rs3093110 was related to promoter histone marks, motifs changed, selected eQTL, GRASP QTL hits.

## Discussion

In the present study, allele, genotype and haplotype frequencies of five SNPs in the *CYP4F2* gene between IS patients and healthy controls were compared and stratified analyses by age and gender were conducted. We found that three SNPs (rs3093193, rs3093144 and rs12459936) in the *CYP4F2* were associated with IS risk in a Chinese Han population. Rs3093144 and rs12459936 were associated with IS risk in the overall. For the individuals older than 61 years old, rs3093193 and rs3093144 were related to the increased risk of IS, whereas rs12459936 was related to the decreased risk of IS. In addition, we observed that three SNPs (rs3093193, rs3093144 and rs12459936) were associated with the susceptibility to IS in the male. And five SNPs in the *CYP4F2* gene showed strong linkage. These findings suggested that genetic polymorphisms in *CYP4F2* may play an important role in the etiology of IS.

Genetic variation is the molecular basis of human genetic diversity. IS is a complex polygenic disease resulting from the genetic factors and environmental risk factors [23]. Approximately 80% of stroke is IS, and the incidence of IS increases with aging. As a member of the CYP450 superfamily, *CYP4F2* plays a pivotal role in the metabolism of exogenous and endogenous compounds [24]. It is expressed in the liver, heart, lungs, and kidneys, and is involved in leukotriene B4 (LTB4) and 20--hydroxy eicosane arachidonic acid (20-HETE) metabolism [25]. 20-HETE in vivo can depolarize vascular smooth muscle by blocking Ca^2+^ activation and K^+^ channels, resulting in strong vasoconstriction [26]. In hypertensive rat models, increased 20-HETE can lead to oxidative stress and endothelial cell damage, thus increasing the incidence of IS [27]. *CYP4F2* is the main synthetase that catalyzes the generation of 20-HETE from arachidonic acid. Recent studies revealed that *CYP4F2* was associated with the IS risk. H.-Q. Yan et al. [28] conducted a case-control study to identify the association between the selected SNPs in *CYP4F2* gene and the risk of IS. The results showed that the ‘GG’ genotype of *CYP4F2* rs2108622 was correlated with an increased risk of IS. In addition, Colàs-Campàs L et al. [29] found rs2108622 ‘AA’ genotype in *CYP4F2* gene was significantly associated with a risk of early IS in non-valvular atrial fibrillation patients under vitamin K antagonists treatment. However, study based on this *CYP4F2* gene is rare. Hence, our study discussed the relationship between *CYP4F2* and IS risk. We found that three SNPs (rs3093193, rs3093144 and rs12459936) in the *CYP4F2* were associated with IS risk in a Chinese Han population, suggesting *CYP4F2* gene may play an important function in affecting IS.

In addition, it is worth noting that in our results, there were two SNPs (rs3093193 and rs3093144) increased the IS risk and one (rs12459936) decreased the IS risk. However, the haplotype analysis among the three SNPs mentioned above found it was significantly associated with an increased risk of IS (Table 7). For rs12459936, the minor allele ‘T’ when compared to the wild-type allele ‘C’ was protective factor. In turn, the wild-type allele ‘C’ was associated with an increased risk of stroke when compared with the minor allele ‘T’ as reference allele. Thus, based on the positive loci, the haplotype “GCT” was related to elevating risk of IS.

Furthermore, we have compared results with other stroke studies performed in the same population. Lee et al. [30] conducted that a genome-wide association study links small-vessel ischemic stroke to autophagy. And their study focused on Han Chinese in Taiwan. In addition, the study was replicated in an independent Han Chinese population. Imputation analysis also supported the association between three SNPs (rs2594966, rs2594973, rs4684776) in *ATG7* gene and stroke-small-vessel occlusion (SVO). When compared to their study, our sample size was not large enough because of strict recruitment criteria. And the study failed to replicate the results within an independent sample. So that’s a part of what we’re going to do next.

Our study also has some potential limitations. The ethnicity of study subjects was limited to the Han Chinese population. Hence, whether our results could apply to other ethnicities is unclear. Furthermore, our current research is fundamental, functional studies are required to understand function of genetic variants and mechanisms underlying this association.

## Conclusions

To sum up, we firstly provide new evidence for the association between *CYP4F2* variants and IS risk in Han Chinese population, which may support for screening of IS in Han Chinese population and shed light on the mechanism of IS.

## Supplementary information

**Additional file 1: Table S1.** Functional prediction results of selected loci in the database. (DOCX 13 kb)

## Data Availability

The datasets supporting the conclusions of this article are included within the article and its additional file.

## Abbreviations

20-HETE: 20--hydroxy eicosane arachidonic acid
CI: Confidence interval
CT: Computed tomography scans
*CYP4F2*: Cytochrome P450 family 4 subfamily F member 2
EDTA: Ethylenediamine tetraacetic acid
GWAS: Genome-wide association studies
HWE: Hardy–Weinberg equilibrium
IS: Ischemic stroke
LD: Linkage disequilibrium
MAF: Minor allele frequency
MRI: Magnetic resonance imaging
OR: Odds ratio
SNP: Single nucleotide polymorphism
