# Supplementary material for: Genetic variants in CYP4F2 were significantly correlated with susceptibility to ischemic stroke
Source: BMC Med Genet. 2019 Sep 11;20:155. doi: 10.1186/s12881-019-0888-6 (PMC6737589; doi:10.1186/s12881-019-0888-6)
Supplement: Supplementary file 1 — Additional file 1: Table S1. Functional prediction results of selected loci in the database. (DOCX 13 kb) [file 12881_2019_888_MOESM1_ESM.docx]

Supplementary table 1. Functional prediction results of selected loci in the database.

| SNP | LD | LD | Ref | Alt | Functional | Haploreg |
| --- | --- | --- | --- | --- | --- | --- |
|  | (r^2^) | (D') |  |  | annotation |  |
| rs3093203 | 1 | 1 | G | A | 3'-UTR | Motifs changed,Selected eQTL hits |
| rs3093193 | 1 | 1 | C | G | intronic | Motifs changed,Selected eQTL hits |
| rs12459936 | 1 | 1 | C | T | intronic | Motifs changed,Selected eQTL hits |
| rs3093144 | 1 | 1 | C | T | intronic | Enhancer histone marks,Motifs changed,Selected eQTL hits |
| rs3093110 | 1 | 1 | A | G | intronic | Promoter histone marks,Motifs changed,Selected eQTL,GRASP QTL hits |

SNP: single-nucleotide polymorphism; LD, linkage disequilibrium; Ref, reference allele; Alt, altered allele; UTR, untranslated region; eQTL: expression quantitative trait loci;GRASP: Genome-Wide Repository of Association between SNPs and Phenotypes.
